# Targeting Lung Damage: Amniotic Mesenchymal Stem Cells Mitigate Lipopolysaccharide-Induced Acute Lung Injury via Multiple Signaling Pathways

**DOI:** 10.3390/ijms26052314

**Published:** 2025-03-05

**Authors:** Xinhui Niu, Lina Zhang, Shaoliang Xing, Jinrui Liu, Deming Li, Yating Wang, Yi Wang, Manman Su

**Affiliations:** 1 Department of Regenerative Medicine, School of Pharmaceutical Sciences, Jilin University, Changchun 130021, China; niuxh22@mails.jlu.edu.cn (X.N.); jrliu22@mails.jlu.edu.cn (J.L.); lidm23@mails.jlu.edu.cn (D.L.); wangyt2822@mails.jlu.edu.cn (Y.W.); 2 NMPA Key Laboratory for Quality Control of Cell and Gene Therapy Medicine Products, Northeast Normal University, Changchun 130024, China; thswzln@163.com (L.Z.); xsl323@126.com (S.X.)

**Keywords:** amniotic MSCs, acute lung injury, TLR4/NF-κB/MAPK, apoptosis, EMT

## Abstract

Acute lung injury (ALI) is a life-threatening condition triggered by pneumonia, viral infections, or physical trauma. It manifests clinically as progressive respiratory failure and refractory hypoxemia. Using a lipopolysaccharide (LPS)-induced acute lung injury mouse model, we demonstrated that amniotic mesenchymal stem cells (AMSCs) exhibit robust reparative and anti-inflammatory properties. Our analysis encompassed inflammatory mediators; histological damage; tight junction integrity; epithelial–mesenchymal transition (EMT); and the TGF-β/Smad, TLR4/NF-κB/MAPK, pyroptosis, and apoptosis signaling pathways. Our key results demonstrated that in ALI-afflicted mice, AMSCs exhibited targeted pulmonary tropism, homing in on injured alveolar regions, where they restored the morphology and functionality of damaged tissues and organelles, re-established lung barrier function, and attenuated the aberrantly activated TLR4/NF-κB/MAPK and TGF-β/Smad pathways associated with inflammation. These coordinated mechanisms contributed to pyroptosis, apoptosis, and fibrosis suppression. In conclusion, AMSCs mitigated the inflammatory injury process in ALI mice through multiple mechanisms, thereby supporting the potential development of MSC-based therapeutic strategies.

## 1. Introduction

Acute lung injury (ALI) and acute respiratory distress syndrome (ARDS) arise as consequences of underlying illnesses such as trauma, viral or bacterial pneumonia, and various blood transfusions [1]. The global progression of epidemic pneumonia has resulted in the emergence of ALI/ARDS as a critical public health concern [2]. Aging populations and the unhealthy lifestyle patterns of our current life further aggravate these problems [3,4,5]. Therefore, exploring ALI/ARDS progression and developing a therapeutic approach to minimize harm would be highly advantageous.

Lipopolysaccharide (LPS) usually interacts with immune or endothelial cells by binding to their pattern recognition receptors, such as Toll-like receptors (TLRs), particularly TLR4 [6,7], and activates the downstream TLR4/NF-κB/MAPK signaling pathway, which depends on myeloid differentiation factor 88 (MyD88) signaling [6,8]. NF-κB and MAPK pathway activation—hallmarked by key players in inflammatory signaling—triggers a cascade that significantly boosts pro-inflammatory mediator production, including that of TNF-α, IL-6, and IL-1β [9]. In the meantime, the NF-κB signaling axis centered on p65 phosphorylates upon nuclear translocation, triggering inflammatory pathways that drive NLRP3 upregulation, vesicle proliferation, and GSDM protein activation, culminating in pyroptosis and inflammatory cascade. Furthermore, the MAPK pathway activates transcription factors including β-catenin and Smad2/3 while coordinating pro-apoptotic regulators such as BAD, Bax, and Bid. This dual regulation drives critical cellular processes ranging from apoptosis and differentiation to migration, tissue infiltration, and inflammatory signaling [10,11]. Pyroptosis is distinguished by its heightened inflammatory response and immunogenic potential relative to apoptosis and serves as a critical component of the host defense against microbial pathogens [12].

Epithelial–mesenchymal transition (EMT) is a reversible process involving multiple pathways and is vital in the pathogenesis of organ fibrosis [13]. The key EMT targets, aside from the previously mentioned inflammatory damage, also relate to an important fibrosis pathway. This TGF-β/Smad signaling pathway reportedly inhibits TGF-β/Smad pathway activation and can mitigate pulmonary fibrosis in mice [14,15]. Furthermore, the PI3K/AKT and MAPK signaling pathway inhibition can curb the epithelial cell-induced inflammatory response and repress the TGF-β/Smad and PI3K/AKT/mTOR signaling pathways, thereby prominently inhibiting fibroblasts-to-myofibroblast transformation and reducing pulmonary fibrosis in mice [16].

Mesenchymal stem cells (MSCs) are rich in cytokines and extracellular vesicles that help reduce inflammation and promote tissue repair and regeneration [17]. MSCs reportedly exert favorable regulatory effects on the MAPK signaling pathway [18]. Amniotic mesenchymal stem cells (AMSCs) proliferate faster than adult-derived MSCs and can differentiate into cell types from all three germ layers [19]. In a severe colitis rat model, AMSC transplantation improved colitis by inhibiting monocyte/macrophage activity, lowering inflammatory markers (TNF-α, IL-6, and IL-1β), and preventing NF-κB activation [20]. When AMSCs migrate to damaged kidney tissues, they enhance angiogenesis via the PI3K/AKT/eNOS pathway, inhibit oxidative stress and EMT, and alleviate renal fibrosis [21]. There, AMSCs exhibit their significant potential as therapeutic agents.

In this study, we examined the regulatory and therapeutic effects of AMSCs on ALI to evaluate their potential as a new treatment.

## 2. Results

### 2.1. Homing Effect Directed AMSCs to ALI Mouse Lungs

**Figure 1:**
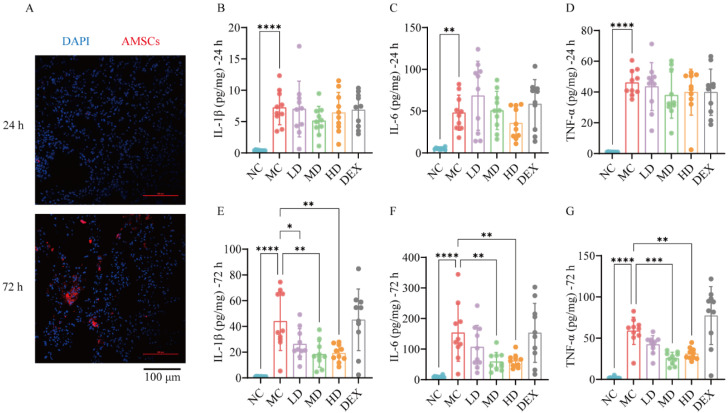
Homing and anti-inflammatory evaluation of AMSCs. (**A**) AMSC localization in ALI mouse lung tissue at 24 and 72 h after injection (laser confocal, scale bar: 100 μm). (**B**–**G**) IL-1β, IL-6, and TNF-α levels in mouse lung tissue at 24 and 72 h. (ELISA, n = 10, One-way ANOVA with Dunnett’s post hoc test, * *p*  <  0.05, ** *p*  <  0.01, *** *p*  <  0.001, **** *p* <  0.0001.) Abbreviations: ALI, acute lung injury; AMSCs, amniotic mesenchymal stem cells; NC, normal control group; MC, model control group; LD, low-dose group; MD, medium-dose group; HD, high-dose group; DEX, dexamethasone.

Stem cell therapy exploits the migratory, regenerative, and paracrine properties of stem cells. These cells migrate to injury sites in response to injury-derived signals, where they exert their therapeutic effects. Figure 1A demonstrates distinct temporal accumulation patterns of AMSCs in lung tissues, with minimal detection at 24 h post-transplantation, contrasting sharply against the substantial cellular engraftment observed at 72 h. This time-dependent biodistribution profile reveals AMSCs’ targeted migration capacity toward LPS-injured pulmonary regions, where subsequent paracrine activity mediated tissue repair processes.

### 2.2. AMSCs Reduced Inflammatory Factors in ALI

Figure 1B–D reveal a significant elevation in pro-inflammatory cytokines (IL-1β, IL-6, TNF-α) in the MC versus NC groups at 24 h post-injury (*p* < 0.01, *p* < 0.0001), confirming successful ALI induction. Notably, neither the AMSC-treated nor DEX groups showed significant inflammatory modulation at this early phase. The longitudinal analysis in Figure 1E–G demonstrates marked reductions (*p* < 0.05~*p* < 0.001) of these cytokines in the AMSC groups at 72-h post-treatment. In summary, the use of AMSCs can reduce the severity of acute inflammation and inhibit the release of pro-inflammatory substances in the lungs during ALI.

### 2.3. AMSCs Mitigated Pathological Lung Injury in a Murine Model of ALI

**Figure 2:**
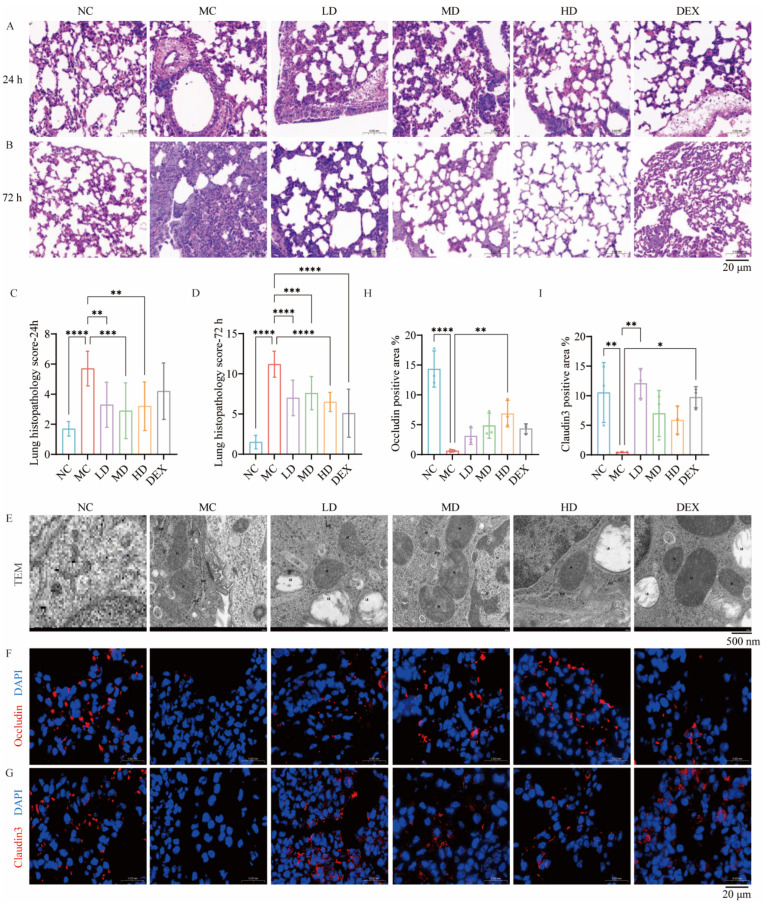
AMSCs reduced ALI-induced lung injury, organelle damage, and tight junction disruption. (**A**,**B**) Lung tissue morphology of mice determined by H&E staining (scale bar: 20 μm). Statistical analysis of pathological scores at 24 h (**C**) and 72 h (**D**). (n = 10, One-way ANOVA with Dunnett’s post hoc test, ** *p*  <  0.01, *** *p*  <  0.001, **** *p* <  0.0001). (**E**) Transmission electron microscope images showing the changes in organelles from different groups (scale bar: 500 nm). (**F**–**I**) Immunofluorescence and quantification of Occludin and Claudin3. (Target protein area/total tissue area*%, scale bar: 20 μm, n = 3, One-way ANOVA with Dunnett’s post hoc test, * *p*  <  0.05, ** *p*  <  0.01, **** *p* <  0.0001.) Abbreviations: ALI, acute lung injury; AMSCs, amniotic mesenchymal stem cells; NC, normal control group; MC, model control group; LD, low-dose group; MD, medium-dose group; HD, high-dose group; DEX, dexamethasone.

The histopathological analysis in Figure 2A demonstrates that after 24 h, the lung tissue of the MC group exhibited increased granulocyte infiltration, alveolar wall thickening, and widened septae. The lumen contained erythrocytes and multifocal alveolar hemorrhage, along with a few detached epithelial cells, granulocytes, and cell debris in the narrow bronchioles. The specimens also exhibited a minor presence of moderate swelling around the blood vessels and a loosely organized arrangement of connective tissue. After AMSC treatment, tissue hemorrhage and edema lessened, but the connective tissue remained loose, granulocyte infiltration showed little improvement, and cell necrosis persisted. These findings suggested that AMSC therapy had been insufficient after 24 h.

After 72 h of the ALI process (Figure 2B), the lung tissue injury was further aggravated in the MC group. An examination of the DEX group revealed occasional vascular swelling, sparse connective tissue with minimal lymphocytes, and moderate to severe thickening and widening of the air sac walls, as well as variable air sac diameters. Therapy with AMSCs significantly reduced the alveolar wall thickening caused by acute injury. Additionally, it could reduce epithelial cell hyperplasia, inflammatory cell infiltration, hemorrhagic edema, and fine bronchial hyperplasia, while restoring alveolar morphology. These findings indicated that AMSCs significantly improved lung pathology in ALI mice; restored the integrity of the epithelial–endothelial barrier; reduced the release of inflammatory mediators, hemorrhage, and edema; and facilitated the gradual recovery of tissue and organ function.

The comparative histopathological scoring in Figure 2C,D revealed that the AMSC-treated groups exhibited significantly lower injury scores than the MC controls at both 24 h (*p* < 0.01, *p* < 0.001) and 72 h (*p* < 0.001, *p* < 0.0001) post-intervention. The MC group showed progressive lesion exacerbation, maintaining higher scores versus NC group (*p* < 0.0001), with 72 h pathology severity exceeding 24 h levels. This temporal progression, combined with parallel inflammatory cytokine reductions at 72 h, established the later timepoint as biologically relevant for subsequent therapeutic mechanism evaluations.

### 2.4. AMSCs Facilitated the Restoration of Organelle Architecture Following ALI

The transmission electron microscopy analysis in Figure 2E revealed distinct ultrastructural preservation by AMSCs in ALI-affected lungs. Compared with the NC group, the MC group II alveolar epithelial cells exhibited evident damage, characterized by uneven electron density in the intracellular matrix, localized lysis, and partial swelling of organelles. The nuclei appeared irregularly shaped (N). The mitochondria (M) showed enlargement and abnormalities, including uneven electron density in the intramembrane matrix, localized vacuolation, and cristae disruption. The rough endoplasmic reticulum (RER) showed localized dilation, membrane lysis, fracture, and ribosome degranulation on its surface. There were fewer lamellar bodies (LB), and the lamellar structures showed lysis and emptying.

A comparative ultrastructural analysis revealed that dexamethasone treatment partially restored cellular architecture in injured lungs, showing reconstitution of plasma and mitochondrial membranes with normalized intracristal matrix electron density, though persistent epithelial defects remained. AMSC administration demonstrated comprehensive cytoprotection, achieving near-normal mitochondrial cristae patterning, endoplasmic reticulum membrane integrity, and nuclear contour restoration alongside significant epithelial repair. These morphological improvements correlated with enhanced cellular functional competence and attenuated inflammatory pathology during acute lung injury progression.

### 2.5. AMSCs Restored Tight Junction Integrity Compromised During ALI

Quantitative analyses revealed marked reductions in Occludin (*p* < 0.01) and Claudin3 (*p* < 0.0001) expression accompanied by elevated alveolar permeability in the MC group specimens. However, the expression levels of Occludin and Claudin3 were partially recovered in the HD group (*p* < 0.01, Occludin) and LD group (*p* < 0.01, Claudin3) compared to the model group (Figure 2F,H and Figure 2G,I). In conclusion, AMSC treatment enhances tight junction integrity between lung cells during acute inflammation, thereby restoring tissue morphology and function.

### 2.6. AMSCs Reduced Collagen Deposition and Fibrosis Following ALI

**Figure 3:**
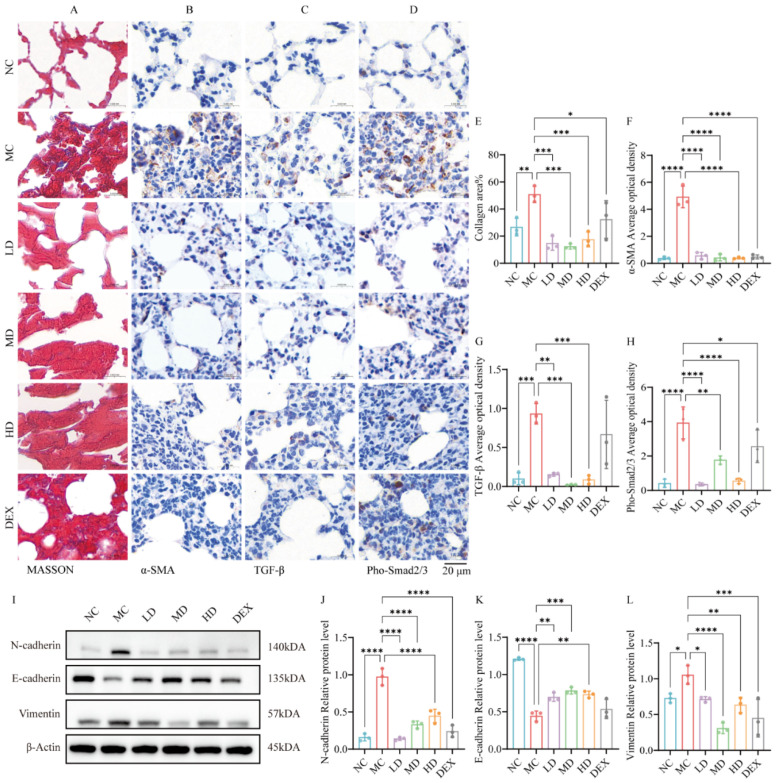
AMSCs attenuated the process of collagen deposition and fibrosis in ALI. (**A**) Masson staining of mouse lung tissue. (**B**–**D**) Immunohistochemical staining analysis of α-SMA, TGF-β and Pho-Smad2/3. (**E**) The area percent of each image that were stained positive for collagen was quantified. (**F**–**H**) Average optical density values for α-SMA, TGF-β and Pho-Smad2/3; average optical density (AOD) (%Area) = integrated optical density (IOD)/effective target area (Area) (scale bar: 20 μm). (**I**) Western blot to measure the levels of epithelial–mesenchymal transition proteins. (**J**–**L**) Quantification of the levels of proteins in (**I**). (n = 3, One-way ANOVA with Dunnett’s post hoc test, * *p*  <  0.05, ** *p*  <  0.01, *** *p*  <  0.001, **** *p* <  0.0001.) Abbreviations: ALI, acute lung injury; AMSCs, amniotic mesenchymal stem cells; NC, normal control group; MC, model control group; LD, low-dose group; MD, medium-dose group; HD, high-dose group; DEX, dexamethasone.

The histochemical analysis in Figure 3A,E demonstrated substantial attenuation of fibrotic collagen accumulation in both the AMSC-treated and DEX groups compared to the model controls (*p* < 0.05, *p* < 0.001), with Masson’s trichrome staining revealing significantly reduced blue-stained collagen fiber deposition in the therapeutic groups versus the MC specimens.

Furthermore, immunohistochemical evaluation in Figure 3B,F revealed marked increase in α-SMA expression in the MC group compared with the NC group (*p* < 0.0001); α-SMA expression was significantly decreased in both the AMSC and DEX-treated groups compared with the MC group (*p* < 0.0001).

The TGF-β/Smad pathway serves as a central mediator of fibrogenesis across multiple organ systems. As shown in Figure 3C,D,G,H, by IHC staining of lung tissues from ALI mice, TGF-β and Pho-Smad2/3 expressions were significantly increased in the MC group compared to the NC group (*p* < 0.001, *p* < 0.0001); in the AMSC-treated groups compared to the MC group, TGF-β and Pho-Smad2/3 expressions were significantly decreased (*p* <  0.01~*p* < 0.0001). EMT is the underlying cause of fibroblast and alveolar epithelial cell proliferation in various lung diseases [22]. The western blot analysis in Figure 3I–L demonstrates that both AMSC and dexamethasone therapies suppressed mesenchymal transition markers (*N*-cadherin: *p* < 0.0001; Vimentin: *p* < 0.05~*p* < 0.0001) while reconstituting E-cadherin expression (*p* < 0.01, *p* < 0.001) in injured pulmonary tissue. This coordinated modulation of epithelial–mesenchymal plasticity indicates that AMSC-mediated attenuation of fibrogenesis occurs through the dual suppression of the TGF-β/Smad signaling axis and EMT progression.

### 2.7. AMSCs Downregulated TLR4/NF-κB/MAPK Pathway Activation in ALI

**Figure 4:**
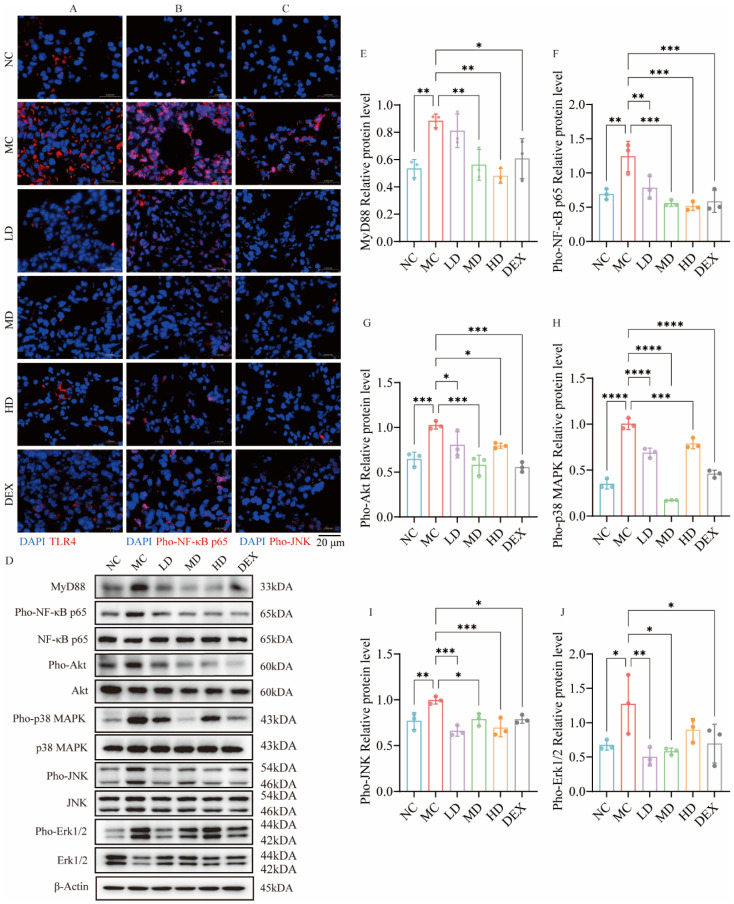
AMSCs downregulated TLR4/NF-κB/MAPK pathway activation in ALI. (**A**–**C**) Immunofluorescence staining was conducted for TLR4, Pho-NF-κB p65, and Pho-JNK in mouse lung tissue. (**D**)The expression of TLR4/NF-κB/MAPK pathway proteins (Western blot analysis). (**E**–**J**) Quantification of the levels of proteins of D. (n = 3, One-way ANOVA with Dunnett’s post hoc test, * *p*  <  0.05, ** *p*  <  0.01, *** *p*  <  0.001, **** *p* <  0.0001, scale bar: 20 μm). Abbreviations: ALI, acute lung injury; AMSCs, amniotic mesenchymal stem cells; NC, normal control group; MC, model control group; LD, low-dose group; MD, medium-dose group; HD, high-dose group; DEX, dexamethasone.

The IF assessment of TLR4 and phosphorylated NF-κB p65 spatial distribution revealed distinct therapeutic effects. Figure 4A demonstrates the pathological upregulation of TLR4 in MC groups compared to NC groups, evidenced by intensified red fluorescence signals. However, TLR4 expression decreased with AMSC and DEX treatment, implying that AMSCs might inhibit NF-κB pathway activation and reduce inflammation via the TLR4 recognition link.

As a pivotal factor of TLR4/MyD88/NF-κB, according to Figure 4B, the MC group exhibited a considerably greater expression of Pho-NF-κB p65 than the NC group. The red fluorescence coincided with the blue light emitted by DAPI in the cell nucleus, suggesting that NF-κB p65 enters the cell nucleus to perform regulatory functions downstream. Then, the expression of Pho-NF-κB p65 was markedly reduced following treatment with AMSCs and DEX compared to the MC group. As shown in Figure 4D–G, the TLR4/MyD88/NF-κB signaling pathway was inhibited to a certain extent after treatment with AMSCs: the MyD88 levels were downregulated by medium- and high-dose AMSCs and DEX compared to the MC group (*p* < 0.05, *p* < 0.01); NF-κB p65 phosphorylation was inhibited under all three groups of dosage administration (*p* < 0.01, *p* < 0.001); in addition, the Akt signaling process as also further activated by LPS, stimulating NF-κB p65 activation; and the results showed that the phosphorylation process of Akt was also significantly inhibited by AMSCs and DEX (*p* < 0.05, *p* < 0.001).

The immunofluorescence analysis revealed intracellular pho-JNK expression, with Figure 4C demonstrating reduced fluorescence intensity in both the AMSC-treated and DEX groups. The Western blot results in Figure 4D,H–J showed significant inhibition of p38 and JNK phosphorylation following these treatments (*p* < 0.05, *p* < 0.001, *p* < 0.0001). At the same time, low/medium-dose AMSCs and dexamethasone additionally suppressed Erk1/2 phosphorylation (*p* < 0.05, *p* < 0.01). These findings collectively indicate AMSC-mediated suppression of the TLR4/NF-κB/MAPK signaling axis in ALI-affected pulmonary tissue.

### 2.8. AMSCs Mitigated Pyroptosis and Apoptosis Triggered by ALI

**Figure 5:**
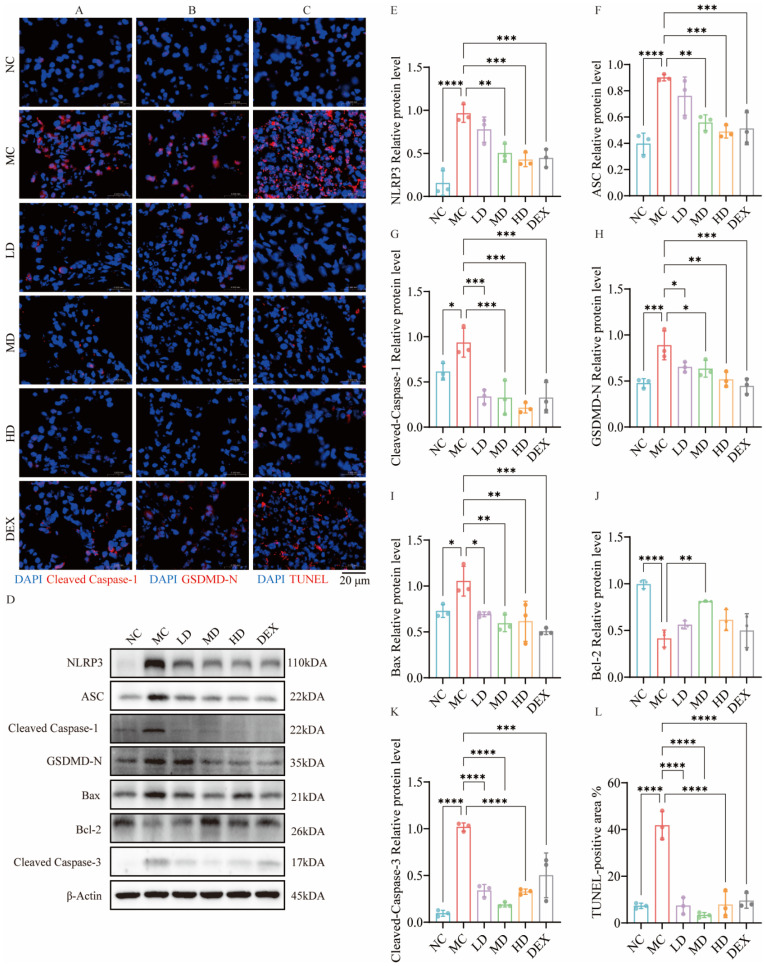
AMSCs reduced pyroptosis and apoptosis in the lung tissue of ALI mice. (**A**,**B**) Immunofluorescence staining was performed to detect Cleaved Caspase-1 and GSDMD-N in mouse lung tissue. (**C**) TUNEL-positive cells stained with TUNEL in ALI mice (scale bar: 20 μm). (**D**) Western blot analysis of the expression levels of proteins involved in pyroptosis and apoptosis pathways. (**E**–**K**) Western blot analysis of D. (**L**) The percentage of TUNEL-positive area was calculated as the ratio of TUNEL-positive areas to total tissue areas. (n = 3, One-way ANOVA with Dunnett’s post hoc test, * *p*  <  0.05, ** *p*  <  0.01, *** *p*  <  0.001, **** *p* <  0.0001.) Abbreviations: ALI, acute lung injury; AMSCs, amniotic mesenchymal stem cells; NC, normal control group; MC, model control group; LD, low-dose group; MD, medium-dose group; HD, high-dose group; DEX, dexamethasone.

As shown in Figure 5, Cleaved Caspase-1 and GSDMD-N were significantly attenuated in both the AMSCs and DEX groups. Meanwhile, both AMSCs and dexamethasone had a significant inhibitory effect on the expression of NLRP3 and ASC (*p* < 0.01, *p* < 0.001). In conclusion, AMSCs significantly inhibited the pyroptosis signaling pathway in the lung tissues of ALI mice.

In Figure 5C,L, compared to the MC group, both AMSCs and dexamethasone treatments significantly reduced the percentage of TUNEL-positive cells (*p* < 0.0001), indicating effective suppression of tissue apoptosis. Furthermore, AMSC treatment significantly downregulated Bax expression and suppressed Cleaved Caspase-3 formation compared to the MC group (*p* < 0.05, *p* < 0.01, *p* < 0.0001). Additionally, AMSC administration at a moderate dose notably upregulated the anti-apoptotic protein Bcl-2 (*p* < 0.01). These findings collectively demonstrate that AMSCs effectively attenuate apoptosis in ALI-affected lung tissues through the coordinated regulation of both pro-apoptotic and anti-apoptotic pathways.

## 3. Discussion

TLR4 is an LPS-specific receptor on the endothelial surface of immune cells, such as monocytes and macrophages [23]. LPS activates downstream kinases in the MAPK and NF-κB signaling pathways via the MyD88-dependent TLR4 pathway, leading directly to apoptosis and pyroptosis [24,25]. Inhibition of the NF-κB and MAPK pathways, two classical inflammatory signaling pathways, helps suppress the production of inflammatory factors. Studies have shown that the downregulation of TLR4/NF-κB/MAPK cascade signaling is fundamental to MSC resistance to cell death and inflammation [26,27]. Our data revealed that AMSCs suppressed TLR4/MyD88 signaling, blocking the nuclear translocation of phosphor-NF-κB p65 and subsequent NLRP3 upregulation, which collectively inhibited Caspase-1 cleavage. This cascade ultimately reduced GSDMD-N production and pyroptosis. In addition, AMSCs attenuated the phosphorylation of p38 MAPK, JNK, and ERK1/2, concurrently decreasing Bax expression and caspase-3 activation while enhancing Bcl-2 levels, thereby downregulating apoptosis. These coordinated effects demonstrated AMSCs’ dual inhibition of the NF-κB and MAPK pathways to mitigate both apoptotic and pyroptotic cell death pathways.

In ALI/ARDS, fibrosis arises from excessive fibroblast growth and ECM build-up, with EMT being pivotal [28]. TGF-β1, a major fibrotic and EMT inducer, drives EMT via the TGF-β/Smad pathway [29,30]. The inhibition of TGF-β1 in animal models reportedly reduces fibrosis, whereas its overexpression in rats leads to severe and progressive lung fibrosis [31]. This experiment showed that AMSCs could reverse the EMT process and attenuate collagen deposition in ALI tissues. This suggests that AMSCs might inhibit this pathway to prevent the fibrotic process associated with ALI.

The paracellular barrier is composed of intercellular tight junctions regulated by the members of the occludin and tight junction protein families [32]. Indeed, during ALI/ARDS, both inflammatory cell infiltration and apoptosis can undermine tight junctions between cells and the alveolar capillary blood membrane, resulting in increased permeability. In our experiments, AMSCs successfully restored the levels of Occludin and Claudin3 in the tissue, which also demonstrated that MSCs have the potential for the multidimensional repair of lung barrier function.

This study has several limitations. First, we drew our conclusions from a small sample size, necessitating larger studies for broader validation before clinical application. Second, the focus on pulmonary changes in ALI mice did not address potential lesions in other organs or therapeutic effects in ALI/ARDS. The biomarkers we used were limited to classic inflammatory mediators, and only data from a single lung tissue homogenate sample were available, representing only a small subset of possible biomarkers. Concerning fibrosis-related research, we mainly relied on histological and WB results and did not detect the corresponding biochemical indicators in the tissue fluid. Finally, we based the AMSC dosage on body weight. However, clinical studies should adjust the dosage for individual differences.

## 4. Materials and Methods

### 4.1. Cells

The AMSCs were supplied by Jilin Zhongke Biotechnology Co., Ltd. (Changchun, China), with the cell products certified by the National Institutes for Food and Drug Control (Certification Reports: SH202003882, SH202003883, SH202100043-SH202100046). Joinn New Drug Research Center conducted the intravenous administration safety assessments. All subsequent experiments utilized sixth-passage cells.

### 4.2. Experimental Animals

Male SPF BALB/c mice (aged 7–9 weeks, 22–24 g body weight) were used in this study. All animal experiments were conducted in compliance with protocols approved by the Animal Welfare and Ethics Committee of Jilin University College of Pharmacy (Approval No. 20230088; 20 December 2023).

### 4.3. Creation of ALI Mouse Model

Following a 3-day acclimatization period, BALB/c mice were anesthetized and stabilized in supine position. A midline neck incision exposed the trachea, through which 20 μL/10 g body weight of LPS solution (2 mg/mL, Escherichia coli-derived, Sigma-Aldrich, St. Louis, MI, USA, #0000211692) was administered using a pancreatic-injection needle. The control animals received equivalent PBS volumes. The injection timing was synchronized with respiratory cycles to enhance pulmonary delivery efficacy. Post-injection, the mice underwent 360° axial rotation to optimize LPS distribution before wound closure.

### 4.4. Grouping of Animals and Administration of Medication

The ALI model animals were randomly divided into five experimental groups: model control (MC): 5 mL/kg saline; AMSC therapy: low-dose (LD): 1 × 10^6^ cells/kg; medium-dose (MD): 2 × 10^6^ cells/kg; high-dose (HD): 4 × 10^6^ cells/kg; positive control (DEX): 1 mg/kg dexamethasone. Normal controls (NCs) received an equivalent saline volume. All treatments were administered via tail vein 30 min post-LPS challenge. (The AMSC dosing regimen was established through preliminary safety assessments and pilot studies.)

At 24 and 72 h post-intervention, the animals were anesthetized with sodium pentobarbital (60 mg/kg, i.p.) for sample collection. Following cervical dislocation euthanasia, thoracotomy revealed intact cardiopulmonary complexes. The lung tissues underwent multi-modal processing: a designated lobe was PBS-rinsed and fixed in 10% neutral buffered formalin for histological analyses (H&E, immunohistochemical (IHC) and immunofluorescence (IF) analyses); precise 1 × 1 × 3 mm^3^ segments were preserved in 4% glutaraldehyde (0.1 M PBS, pH 7.4) for ultrastructural transmission electron microscopy evaluation; fresh tissue was homogenized (10% *w*/*v*) in ice-cold saline for biochemical assays; and any residual tissue was snap-frozen in liquid nitrogen and stored at −80 °C for protein analysis.

### 4.5. Homing Detection of AMSCs

The CM-Dil labeling procedure commenced with the preparation of a 1 mg/mL stock solution by dissolving 50 μg CM-Dil in 50 μL DMSO, which was aliquoted and stored at −20 °C protected from light.

For cell labeling, digested P6 AMSCs were pelleted by centrifugation and resuspended in 1 mL PBS containing 5 × 10^6^ cells mixed with 5 μL CM-Dil stock solution. The cell suspension underwent sequential temperature treatment: initial incubation at 37 °C for 5 min followed by 15 min of cooling at 4 °C, after which labeled cells were washed thrice with PBS via centrifugation cycles. These CM-Dil-tagged AMSCs were then administered to ALI mice through tail vein injection. At 24 and 72 h post-injection, lung tissues were harvested, embedded in O.C.T compound, and snap-frozen in liquid nitrogen for cryopreservation. Cryosections were prepared and mounted with DAPI-Fluoromount-G medium. Cellular tracking was accomplished through laser scanning confocal microscopy, where CM-Dil-labeled AMSCs exhibited distinct red fluorescence signals against DAPI-stained nuclei.

### 4.6. Histological Examination

Following fixation in 4% paraformaldehyde, lung tissue specimens underwent standard histological processing including dehydration, paraffin embedding, and sectioning. Serial sections were subjected to H&E staining for morphological evaluation, dual IF and IHC analyses for protein localization, and Masson’s trichrome staining to quantify collagen deposition as a fibrosis indicator. A parallel ultrastructural analysis was conducted through transmission electron microscopy to visualize subcellular architectural changes.

For H&E staining, a pathological scoring of lung tissue inflammation was further conducted in this study, referring to the method of Mikawar [33], as follows: (a) alveolar congestion, (b) hemorrhage, (c) infiltration or aggregation of neutrophils in the air cavity or vascular wall, and (d) widening of the alveolar septum/formation of a hyaline membrane. Each item was scored on a 5-point scale as follows: 0 points: extremely mild damage; 1 point: mild damage; 2 points: moderate damage; 3 points: severe damage; 4 points: very severe damage. The total score was obtained by summing the scores of each indicator.

Regarding the transmission electron microscopy sample preparation, the post-fixed lung tissue was rinsed with 0.1 M phosphate buffer at pH 7.4 and then subjected to fixation with 1% osmium acid at room temperature in the dark, dehydration at room temperature, infiltration and embedding, and polymerization. Ultra-thin sections (60–80 nm) were prepared. Eventually, after staining with 2% saturated uranyl acetate alcohol solution in the dark, staining with 2.6% lead citrate solution was conducted. After drying, the samples were utilized for transmission electron microscopy observation and image acquisition and analysis.

This study employed ImageJ1.53t for comprehensive quantitative analyses, including protein band densitometry in Western Blot, fluorescence signal quantification in immunofluorescence, collagen deposition assessment via Masson’s trichrome staining (calculated as Collagen Area% = [stained collagen area/total tissue area] × 100%), and immunohistochemical evaluation using average optical density (AOD). The AOD metric was derived by normalizing the integrated optical density values to the region of interest area (AOD = IOD/Area), representing the target protein expression levels per unit tissue area.

### 4.7. TUNEL Analysis

To enhance tissue permeability, proteinase K pretreatment was applied to tissue sections prior to incubation with the TUNEL assay kit (Servicebio Co., Ltd., Wuhan, China) for apoptotic cell detection. Nuclei were subsequently counterstained with DAPI (4′,6-diamidino-2-phenylindole) to enable dual fluorescence visualization. Processed samples were analyzed using a fluorescence microscope equipped with appropriate filter sets, and digital images were acquired for a quantitative assessment of apoptosis signals relative to nuclear staining.

### 4.8. Analysis of Levels of Inflammatory Factors

To assess pulmonary inflammatory status, concentrations of the inflammatory cytokines IL-1β, IL-6, and TNF-α in lung tissue homogenates were quantitatively determined using species-specific ELISA kits (Mlbio Co., Ltd., Shanghai, China) according to the manufacturer’s standardized protocols.

### 4.9. Western Blot Analysis

Lung tissue proteins were extracted using a RIPA lysis buffer, quantified via BCA assay, and stored at −20 °C for subsequent analysis. Following SDS-PAGE separation, resolved proteins were electrophoretically transferred onto PVDF membranes. The following antibodies were diluted with primary antibody diluent at a ratio of 1:1000: MyD88, p38 MAPK, pho-p38 MAPK, JNK, pho-JNK, NF-κB p65, pho-NF-κB p65, Erk1/2. Pho-Erk1/2, AKT, pho-AKT, NLRP3, cleaved Caspase-1, GSDMD-N, ASC, Bax, Cleaved Caspase-3, E-cadherin, *N*-cadherin, Vimentin, and β-actin (Cell Signaling Technology, USA). Antibodies were applied to a PVDF membrane separately and incubated overnight at 4 °C with gentle agitation on a shaker. Membranes were subsequently probed with HRP-conjugated secondary antibodies (1:3000 dilution) for 1 h at room temperature, followed by five 5-min TBST washes. The protein bands were visualized with an ECL reagent and then images were recorded.

### 4.10. Statistical Analysis

The results are expressed as the mean ± standard deviation. Statistical evaluation was performed using GraphPad Prism 10. One-way analysis of variance (ANOVA) with Dunnett’s post hoc test was employed for group comparisons. *p* < 0.05 was considered statistically significant.

## 5. Conclusions

Our investigations indicate that AMSCs exert multimodal therapeutic effects against LPS-induced acute lung injury. Key findings indicate that AMSCs (1) suppress inflammatory factors; (2) ameliorate pulmonary hemorrhage, edema, and alveolar wall hyperplasia; and (3) mitigate tissue damage via the dual inhibition of TLR4/NF-κB/MAPK and TGF-β/Smad signaling cascades. Our findings suggested that AMSCs may be a promising multipotent therapy for ALI.

## Data Availability

The data that support the findings of this study are available on request from the corresponding author, M.S., upon reasonable request.
